# Evidence of promiscuous endothelial binding by *P**lasmodium falciparum*‐infected erythrocytes

**DOI:** 10.1111/cmi.12270

**Published:** 2014-02-24

**Authors:** Claudia Esser, Anna Bachmann, Daniela Kuhn, Kathrin Schuldt, Birgit Förster, Meike Thiel, Jürgen May, Friedrich Koch‐Nolte, María Yáñez‐Mó, Francisco Sánchez‐Madrid, Alfred H. Schinkel, Sirpa Jalkanen, Alister G. Craig, Iris Bruchhaus, Rolf D. Horstmann

**Affiliations:** ^1^ Department of Molecular MedicineBernhard Nocht Institute for Tropical MedicineBernhard‐Nocht‐Strasse 7420359HamburgGermany; ^2^ Department of Molecular ParasitologyBernhard Nocht Institute for Tropical MedicineBernhard‐Nocht‐Strasse 7420359HamburgGermany; ^3^ Infectious Disease Epidemiology GroupBernhard Nocht Institute for Tropical MedicineBernhard‐Nocht‐Strasse 7420359HamburgGermany; ^4^ Institute of ImmunologyUniversity Medical Center Hamburg‐EppendorfMartinistrasse 52HamburgGermany; ^5^ Servicio de InmunologíaHospital Universitario de la PrincesaUniversidad Autónoma de MadridC Diego de Leon 6228006MadridSpain; ^6^ Departamento de Biología Vascular e InflamaciónCentro Nacional de Investigaciones CardiovascularesMelchor Fernández Almagro 328029MadridSpain; ^7^ Division of Molecular BiologyThe Netherlands Cancer InstitutePlesmanlaan 1211066 CXAmsterdamThe Netherlands; ^8^ MediCity Research Laboratory and Department of Medical Microbiology and ImmunologyUniversity of Turku and National Institute of Health and WelfareTykistökatu 6A20520TurkuFinland; ^9^ Liverpool School of Tropical MedicinePembroke PlaceLiverpoolL3 5QAUK

## Abstract

The adhesion of infected red blood cells (iRBCs) to human endothelium is considered a key event in the pathogenesis of cerebral malaria and other life‐threatening complications caused by the most prevalent malaria parasite *P**lasmodium falciparum*. In the past 30 years, 14 endothelial receptors for iRBCs have been identified. Exposing 10 additional surface proteins of endothelial cells to a mixture of *P**. falciparum* isolates from three Ghanaian malaria patients, we identified seven new iRBC receptors, all expressed in brain vessels. This finding strongly suggests that endothelial binding of *P**. falciparum* iRBCs is promiscuous and may use a combination of endothelial surface moieties.

## Introduction

Severe malaria caused by infection with *Plasmodium falciparum *is a complex clinical syndrome comprising a number of life‐threatening conditions including cerebral malaria (CM). Several lines of evidence indicate that a major mechanism underlying the pathology of CM is the ‘cytoadherence’ of infected red blood cells (iRBCs) to the endothelium lining the microvasculature of the brain, which may cause disturbances of the cerebral blood flow and metabolism as well as local inflammatory responses (Berendt *et al*., 1994; van der Heyde *et al*., 2006).

Cytoadherence is mediated by the binding of *Plasmodium*‐derived proteins exposed on the surface of iRBCs to defined structures on endothelial cells. One of the major adhesion molecules of the parasite is the *P. falciparum *erythrocyte membrane protein 1 (*Pf*EMP1) (Leech *et al*., 1984). *Pf*EMP1 comprises a great variety of proteins encoded by 60 *var *genes in the individual parasite genome and numerous variants in the parasite population at large. By varying and switching *var *gene expression the parasites show different and changing binding phenotypes, accompanied by antigenic variation (Miller *et al*., 2002).

Until now, 14 endothelial surface structures have been identified to serve as receptors for *P. falciparum* iRBCs, including CD36, intercellular adhesion molecule‐1 (ICAM‐1), vascular cell adhesion molecule‐1 (VCAM‐1), platelet/endothelial cell adhesion molecule (PECAM‐1), neural cell adhesion molecule (NCAM) and endothelial protein C receptor (EPCR). Additionally, chondroitin sulfate A (CSA) expressed on syncytiotrophoblast cells can serve as attachment points for iRBCs in the placenta. Only CD36, ICAM‐1, PECAM‐1, EPCR and CSA have been studied in detail (Serghides *et al*., 2003; Chakravorty and Craig, 2005; Rowe *et al*., 2009; Berger *et al*., 2013; Moxon *et al*., 2013; Turner *et al*., 2013), revealing a unique association of binding to CSA with placental malaria (Duffy and Fried, 2005). For CM it remains unclear whether any single one of the known endothelial structures has a pivotal role. ICAM‐1 was implicated by immunopathological studies of post‐mortem tissues showing colocalization of sequestration with ICAM‐1 expression in the brain (Turner *et al*., 1994) and by an *in vitro *analysis of isolates from CM patients preferentially binding to recombinant ICAM‐1 (Newbold *et al*., 1997). In studies with field isolates from Asia, however, ICAM‐1 binding was not associated with severe malaria (Ockenhouse *et al*., 1991; Udomsangpetch *et al*., 1996). CD36 is the quantitatively most important receptor as almost all non‐placental *P. falciparum* isolates derived from patients bind to it, but a major role of CD36 in cerebral malaria is uncertain because (i) the degree of binding to CD36 by iRBCs isolated from malaria patients did not correlate with severity of the disease (Newbold *et al*., 1997) and (ii) CD36 expression levels on brain endothelial cells was found to be low in CM patients (Turner *et al*., 1994). EPCR is the most recently identified endothelial receptor, which has been implicated in the pathology of severe malaria. While Turner *et al*. showed the direct interaction of EPCR with domain cassettes 8 and 13 of *Pf*EMP1 (Turner *et al*., 2013), Moxon and colleagues revealed a role of EPCR in CM using an entirely different approach (Moxon *et al*., 2013). In post‐mortem studies of children who died of CM, they observed a colocalization of endothelial sites of adherent iRBCs and a loss of EPCR antigens.

For a better understanding of the pathology of CM but also for strategies to develop a vaccine against CM, it is of relevance whether the repertoire of endothelial receptors for iRBC is rather limited or broader than presently recognized. Therefore, we studied 10 surface moieties of the cerebral endothelium for their ability to serve as iRBC receptors. Seven of these showed iRBC binding when a mixture of patient isolates of *P. falciparum *from Ghana, West Africa, was used. This finding strongly suggests that, in contrast to our present understanding, endothelial binding of *P. falciparum *iRBC is highly promiscuous.

## Results and discussion

The tetraspanins CD9 and CD151, multidrug‐resistance protein 1 (MDR1), multidrug resistance‐associated protein 2 (MRP2), the histamine H1 receptor (HRH1), oxidized low‐density lipoprotein receptor 1 (LOX1) and vascular adhesion protein 1 (VAP‐1) as well as truncated forms of tumour necrosis factor receptors 1 (TNFR1) and 2 (TNFR2) and the erythropoietin receptor (EPOR) were expressed in glycosaminoglycan‐deficient Chinese hamster ovary (CHO‐745) cells. All have been shown to be expressed on human brain endothelial cells (Cordon‐Cardo *et al*., 1989; Rossler *et al*., 1992; Salmi *et al*., 1993; Purkiss *et al*., 1994; Sawamura *et al*., 1997; Sincock *et al*., 1997; Dombrowski *et al*., 2001; Kadhim *et al*., 2006; Wosik *et al*., 2007; Medana *et al*., 2009). Tagged with green fluorescent protein (GFP) at their intracellular domains, the proteins were localized to the cell membrane by microscopy, and extracellular exposure was confirmed using non‐permeabilized cells probed with specific antibodies; exceptions were LOX1, which was not GFP labelled but shown to be surface expressed by antibody staining, and MRP2, for which no antibodies or antisera are available directed to extracellular domains but which showed a characteristic GFP fluorescence at the outer cell membrane (Fig. S1). Binding of *P. falciparum* iRBCs was studied using trophozoite stages of the laboratory strain FCR3 and of isolates from three Ghanaian patients with severe falciparum malaria. The patients' samples were pooled, expanded, frozen in aliquots and used as such in order not to introduce additional artefacts by cloning and prolonged *in vitro* cultivation. Cells transfected with CD36, which has been found to bind iRBC from almost all *P. falciparum* isolates (Newbold *et al*., 1997; Rowe *et al*., 2009), were used as positive controls for adhesion.

**Figure 1:**
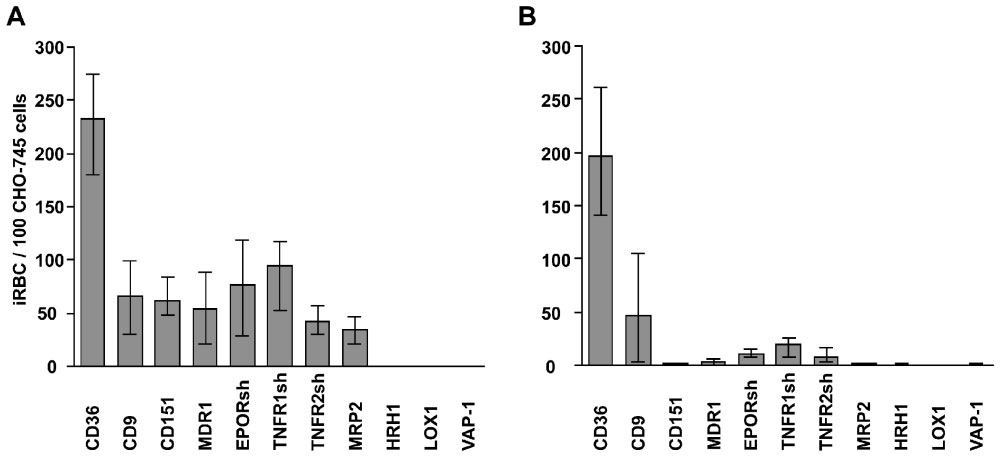
Binding of *P**. falciparum* iRBCs to endothelial surface proteins expressed on CHO cells. Binding of RBCs infected with a pool of *P**. falciparum* isolates from Ghanaian patients (A) or *P**. falciparum* laboratory strain FCR3 (B) to CHO‐745 cells expressing CD36, CD9, CD151, MDR1, EPORsh, TNFR1sh, TNFR2sh, MRP2, HRH1, LOX1 and VAP‐1 respectively. Bars indicate median iRBC numbers specifically bound to 100 CHO cells as determined by microscopic inspection of 500 CHO cells in three independent experiments, each performed in triplicate.

**Figure 2:**
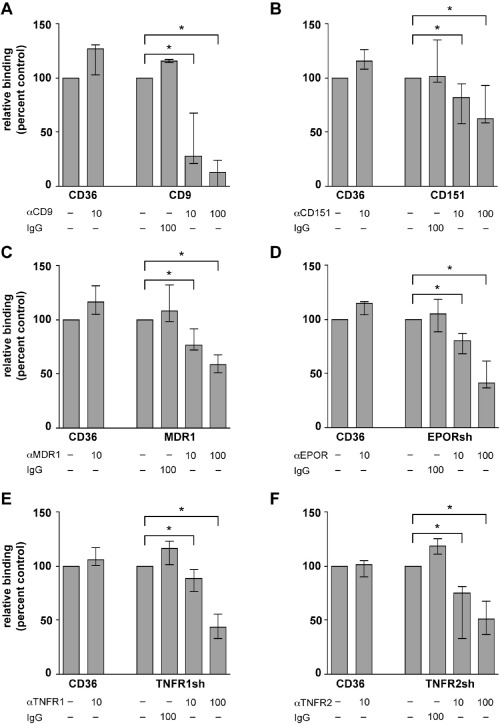
Inhibition of *P**. falciparum* iRBC binding by antibodies to endothelial surface proteins. Inhibition of iRBC binding infected with a mixture of *P**. falciparum* patient isolates to CHO cells expressing the endothelial surface proteins CD9 (A), CD151 (B), MDR1 (C), EPORsh (D), TNFR1sh (E) and TNFR2sh (F) by antibodies to endothelial surface proteins as indicated. Binding to cells expressing CD36 (CD36) and binding inhibition by non‐specific mouse IgG2a (IgG) were used for control. Binding in the presence of 10 μg ml^−1^ (10) and 100 μg ml^−1^ (100) antibodies is compared to the binding in the absence of antibodies (−). Median percentages with ranges of three independent experiments are shown, each performed in triplicate. Asterisks mark significant differences with *P* < 0.05.

Significant binding of the pool of field isolates to CHO‐745 cells transfected with CD9, CD151, MDR1, MRP2, or truncated forms of EPOR (EPORsh), TNFR1 (TNFR1sh) and TNFR2 (TNFR2sh) was detected (Fig. 1A) whereas the laboratory strain FCR3 showed marginal binding, if any (Fig. 1B). Neither FCR3 nor the field isolates caused iRBC binding to cells transfected with HRH1, LOX1 or VAP‐1 (Fig. 1A and B) while both bound most strongly to the positive control CD36. Inhibition assays were performed with purified immunoglobulin G from rat or rabbit antisera (αCD9, αCD151, αEPOR, αTNFR1) and mouse monoclonal antibodies (αMDR1, αTNFR2) respectively. All showed statistically significant inhibition in a dose‐dependent manner, confirming the specificity of the binding reactions of MDR1, EPOR, TNFR1 and TNFR2 (Fig. 2). This interpretation is less straightforward for the tetraspanins CD9 and CD151 because their major function is to assemble multi‐component complexes on cellular surfaces, so‐called tetraspanin‐enriched microdomains, which may enhance the binding capacity of other cellular receptors (Barreiro *et al*., 2005). These might include NCAM and other, as yet uncharacterized iRBC receptors which may be present on non‐transfected CHO‐745 cells (Andrews *et al*., 2005; Pouvelle *et al*., 2007).

We conclude from our data that the binding behaviour of *P. falciparum* iRBCs is highly promiscuous. If our findings obtained with a field isolate pool can be extrapolated to the plethora of parasite clones in the field, it would suggest that *P. falciparum* may take advantage of many endothelial surface moieties for the adhesion of iRBCs. As indicated by the binding to MDR1 and MRP2, this may include structures which physiologically do not function as cellular receptors, as has first been noted for chondroitin sulfate A (Rogerson *et al*., 1995). Possibly the knob‐like protrusions formed by the parasite at the iRBC surface allow binding to surface structures that are less prominent than genuine receptors.

Certain lines of evidence have indicated that ICAM‐1 and EPCR may play a pivotal role in the adhesion of iRBC in cerebral vessels and, therefore, in the pathology of CM (Turner *et al*., 1994; 2013; Newbold *et al*., 1997; Moxon *et al*., 2013). Our data indicate, however, that multiple ‘receptors’ may participate in the binding of iRBCs to cerebral endothelia as all seven iRBC receptors identified here have been shown to be expressed on endothelial cells of the human brain (Cordon‐Cardo *et al*., 1989; Rossler *et al*., 1992; Sincock *et al*., 1997; Dombrowski *et al*., 2001; Kadhim *et al*., 2006; Wosik *et al*., 2007; Medana *et al*., 2009). In fact, it is conceivable that the brain may be a common site of iRBC adhesion and sequestration because, constituting the blood–brain barrier, the cerebral endothelium is extraordinarily rich in receptor and transporter molecules offering a broad variety of binding sites for iRBCs, which all in all are armed with an even broader variety of adhesion ligands (Miller *et al*., 2002). Thus, our findings could question present concepts of developing anti‐disease vaccines in malaria based on the inhibition of cerebral iRBC adhesion (Smith *et al*., 2000).

Except for the adhesion itself, functional implications of the newly discovered binding reactions have not been the objective of our study and, to this end, any considerations must remain highly speculative. Nevertheless, some comments appear justified.

Several mechanisms have been proposed of how endothelial adhesion of *P. falciparum* iRBC might contribute to organ failure and, in particular, to CM. One is the concept of a mechanical occlusion of blood flow by a ‘sludge’ of adherent iRBCs, which bind additional iRBCs as well as uninfected RBCs and platelets. More recently, it has been discussed that microcirculatory disturbances could also be initiated by pro‐inflammatory cytokines, which are released systemically or locally through adherent iRBCs (Clark *et al*., 2006). These and additional mechanisms might involve the deregulation of endothelial receptor signalling and the damaging of endothelial barrier functions by iRBCs (Miller *et al*., 2002). Therefore, an elucidation of the role of the adhesion process in the development of severe malaria requires further studies.

CD9 and CD151 belong to the protein family of tetraspanins, which have been found to be involved in a variety of cellular functions including proliferation, adhesion, migration, cell fusion as well as binding and processing of pathogens. They may contribute to the cellular attachment of certain bacteria (Green *et al*., 2011) and have been shown to be utilized by *P. falciparum* sporozoites (Silvie *et al*., 2003) and several viruses for host cell entry (Pileri *et al*., 1998; Yoshida *et al*., 2008). A physical association of CD9 and CD151 with ICAM‐1 and VCAM‐1 has been reported to play a crucial role in the firm adhesion of leucocytes during extravasation (Barreiro *et al*., 2005). Both ICAM‐1 and VCAM‐1 are well‐known iRBC receptors (Rowe *et al*., 2009). Therefore, direct binding of iRBCs to the tetraspanins may further contribute to a deregulation of leucocyte trafficking, which may play an important role in the pathogenesis of CM (Miller *et al*., 2002). Furthermore, CD9 is expressed on platelets (Miao *et al*., 2001), and clumping of iRBCs and platelets has been found associated with CM (Pain *et al*., 2001). Thus, similar to CD36, CD9 may contribute to the clumping of iRBCs and platelets and to the binding of such aggregates to the vessel wall.

Cerebral sequestration of iRBCs has been found to be positively correlated with the expression of EPOR in brain vessels (Medana *et al*., 2009), which is in good agreement with our finding of iRBC binding to EPOR. While the function of EPOR on brain endothelial cells is not yet fully understood, it has been reported that EPOR acts as an active transporter of erythropoietin (EPO) across the blood–brain barrier (Brines *et al*., 2000) and that EPO inhibits the permeability of the blood–brain barrier (Casals‐Pascual *et al*., 2008). In addition, elevated EPO concentrations in the plasma of children with CM have been found associated with a reduced risk of neurological sequelae (Martinez‐Estrada *et al*., 2003). Therefore, EPO is being discussed as a neuroprotective agent and an adjuvant treatment for severe malaria. Thus, binding of iRBCs to EPOR might contribute to the pathology of CM not only by disturbing the cerebral microcirculation but also by affecting the integrity of the blood brain barrier through blocking of EPO binding sites.

Several lines of evidence indicate that TNF plays an important role in the development of CM (Grau *et al*., 1989; Kwiatkowski *et al*., 1990), and its two receptors TNFR1 and TNFR2 are most likely to mediate this effect. It is reasonable to assume that direct binding of iRBCs to these receptors could in one way or another modify TNF activities, including the induction and upregulation of ICAM‐1 and other endothelial iRBC receptors (Mackay *et al*., 1993; Lucas *et al*., 1997; Stoelcker *et al*., 2002).

MDR1 and MRP2 are ATP‐Binding‐Cassette (ABC) transporters that may play a critical role in neuroprotection by eliminating a wide range of toxic compounds from the brain (Begley, 2004). Both proteins span the cell membrane multiple times with their functional ATP‐binding cassettes located at the cytosolic side. Neither transporter has so far been recognized to serve as receptors nor have they been implicated in the attachment or invasion of pathogens. Therefore, our data on MDR1 and MRP2 show that *P. falciparum* iRBC may use an extraordinarily broad variety of endothelial surface structures for adherence.

In conclusion, it is conceivable that the additional endothelial receptors for iRBC described here may contribute to the pathology of severe malaria and, in particular, of CM through a number of mechanisms, which, besides the mechanical obstruction of blood flow, possibly include interference with important functions of brain endothelial cells.

## Experimental procedures

### Ethics statement

Patient isolates were collected from three children diagnosed with severe falciparum malaria of a case–control study conducted in Kumasi, Ghana (May *et al*., 2007). The study had been approved by the Committee for Research, Publications and Ethics of the School of Medical Sciences, Kwame Nkrumah University of Science and Technology, Kumasi, Ghana (reference number CHRPE/01/11). All procedures were explained to parents or guardians of the participating children in the local language, and written or thumb‐printed informed consent was obtained (May *et al*., 2007).

Animal use for antisera generation was carried out in strict accordance with the recommendations of the European Union guidelines for the handling of laboratory animals (Directive 2010/63/EU of the European Parliament and the Council of 22 September 2010 on the protection of animals used for scientific purposes). The procedures were approved by the Behörde für Gesundheit und Verbraucherschutz der Stadt Hamburg according to §10a TierSchG (German Protection of Animals Act).

### *P**. falciparum* isolates

*Plasmodium falciparum* strain FCR3 was obtained from Dr Mo‐Quen Klinkert. *In vitro* cultivation of *P. falciparum* was carried out in human Rh‐positive RBCs of blood group 0 according to standard protocols (Trager and Jensen, 1976). Patient isolates were cryopreserved, adapted to *in vitro* cultivation and pooled. Parasite growth was synchronized using 5% sorbitol (Lambros and Vanderberg, 1979).

### Protein expression by CHO cells

Chinese hamster ovary cells defective in glycosaminoglycan biosynthesis (CHO‐745) were obtained from American Type Culture Collection (Esko *et al*., 1985) and grown in Ham's F‐12 media (PAA Laboratories) supplemented with 10% fetal calf serum, penicillin and streptomycin. Expression constructs were generated according to established protocols. cDNA of CD9 and CD151 were cloned into pEGFP‐N1 (Clontech Laboratories), MDR1, MRP2 and HRH1 (Missouri S&T cDNA Resource Center) into pAcGFP‐N1 (Clontech Laboratories), and cDNA of VAP‐1 and LOX1 (ImaGenes GmbH) into pAcGFP‐C1 (Clontech Laboratories) and pcDNA3.1(+) (Invitrogen) respectively. For EPOR, TNFR1 and TNFR2 (ImaGenes GmbH) intracellularly truncated forms (EPORsh, TNFR1sh and TNFR2sh respectively) were generated by cloning amino acids 1–336 of EPOR (using primers 1 and 2), amino acids 1–252 of TNFR1 (using primers 3 and 4) and amino acids 1–307 of TNFR2 (using primers 5 and 6) into pAcGFP‐N1.

Primer 1: 5′‐AAT AAG CTT ACC ACC ATG GAC CAC CTC GGG GCG‐3′
Primer 2: 5′‐ATT CCG CGG TGA CTC TGA GAG GAC TTC CAG‐3′
Primer 3: 5′‐AAT CTC GAG ACC ACC ATG GGC CTC TCC ACC GTG‐3′
Primer 4: 5′‐CAA TCC GCG GTG TCG ATT TCC CAC A‐3′
Primer 5: 5′‐AAT CTC GAG ACC ACC ATG GCG CCC GTC GCC GTC‐3′
Primer 6: 5′‐CAA TCC GCG GCT TAT CGG CAG GCA A‐3′

CHO‐745 cells were transfected using Lipofectamine 2000 (Invitrogen) complexed with pAcGFP1‐N1, pEGFP‐CD9, pEGFP‐CD151, pAcGFP‐MDR1, pAcGFP‐EPORsh, pAcGFP‐TNFR1sh, pAcGFP‐TNFR2sh, pAcGFP‐MRP2, pAcGFP‐HRH1, pcDNA3.1‐LOX1 and pAcGFP‐VAP‐1, respectively, according to the protocol of the manufacturer. Two days after transfection, 0.7 mg ml^−1^ G418 (Biochrom AG) was added to the cultures. G418‐resistant cells were harvested and subjected to FACS sorting using a BD FACSAria cell sorter (BD Biosciences). Single‐cell clones were maintained in 96‐well plates and screened for plasma membrane expression of recombinant proteins.

### Antibodies

The following antibodies were used for immunofluorescence and inhibition studies: Mouse αMDR1 (UIC2, Millipore), αTNFR2 (MAB226, R&D Systems), αHRH1 (MAB4726, R&D Systems), αVAP‐1 (TK8‐14, Santa‐Cruz) and purified mouse IgG2a (MOPC‐173, Biozol) were purchased. Immune sera against human CD9, CD151, EPOR, TNFR1 and LOX1 were generated by cDNA immunization. Full‐length cDNA fragments of CD9, CD151 and LOX1 and truncated forms of EPOR (EPORsh) and TNFR1 (TNFR1sh) were cloned into pcDNA3.1(+) vector (Invitrogen) and coated onto 1 μm‐gold particles (Bio‐Rad). cDNA of CD9, EPORsh and TNFR1sh was ballistically injected into rats and cDNA of CD151 and LOX1 into rabbits at a pressure setting of 400 psi. Gene gun immunization was repeated three times at 6‐week intervals (Koch‐Nolte *et al*., 2005). The immune sera were tested by immunofluorescence staining of CHO cells transfected with CD9, CD151, EPORsh, TNFR1sh and LOX1 respectively. IgG‐antibodies were purified from sera by Protein G Sepharose 4 Fast Flow according to the protocol of the manufacturer (GE Healthcare) and referred to as αCD9, αCD151, αEPOR, αTNFR1 and αLOX1.

### Immunofluorescence studies

CHO‐745 cells were grown on coverslips and fixed with 4% para‐formaldehyde. Surface‐exposed CD9, CD151, MDR1, EPORsh, TNFR1sh, TNFR2sh, HRH1, LOX1 and VAP‐1 were labelled using the respective antibodies and secondary antibodies conjugated to Alexa‐Fluor‐594 (Invitrogen). Nuclei were stained with DAPI at 0.1 μg ml^−1^ (Sigma). Fluorescence‐labelled cells were inspected using a Zeiss Axioskop2 plus microscope (Carl Zeiss AG). Mock‐transfected cells did not react with any of the specific antisera, and CHO cells transfected with endothelial receptors incubated with pre‐immune sera or with secondary antibodies alone did not show fluorescence in any experiment.

### Static iRBC adhesion assays

Stably transfected CHO‐745 cells were grown on 13 mm coverslips and maintained in 24‐well plates. CHO‐745 cells were seeded at a density of 15 000 cells per well and grown for 48 h before assays. For inhibition assays, CHO‐745 transfectants were pre‐incubated with antibodies at 37°C for 30 min. Trophozoite stage parasite cultures at 1% haematocrit and 4% parasitaemia in binding medium (RMPI medium 1640 supplemented with 2% glucose, pH 7.2) were applied to CHO‐745 transfectants and incubated at 37°C for 1 h with gentle mixing at 10 min intervals. Afterwards, unbound erythrocytes were gently removed by washing with binding medium, and the cells were fixed with 1% glutaraldehyde in PBS at room temperature for 1 h. Cells on coverslips were stained with Giemsa, and the numbers of adherent iRBCs per 500 CHO‐745 cells were counted using a light microscope. Assays were performed in triplicate in three independent experiments, in each case determining the binding of iRBCs to mock‐transfected CHO‐745 and CHO‐745‐CD36 for reference. The numbers of iRBCs bound to mock transfectants were subtracted from the numbers of iRBCs bound to CHO‐745 cells expressing recombinant proteins. If this resulted in a negative value, the number of specifically bound iRBCs was set to zero. On average, 25.9 ± 34.9 iRBCs bound to 100 mock‐transfected CHO‐745 cells, which could not be inhibited by adding any of the antibodies to the endothelial surface proteins studied. Data are presented as median with range. For inhibition assays, binding in the presence of antibodies was expressed as percentage of binding in the absence of antibodies. For statistical evaluations the non‐parametric Mann–Whitney *U* test was used, whereby two‐sided *P*‐values of < 0.05 were considered significant (*n* = 6).

## Supplementary Material

**Fig. S1.** Antibody staining of recombinant proteins expressed on the surface of CHO‐745 cells. Immunofluorescence analyses (IFA) with non‐permeabilized CHO‐745 cells confirm surface localization of the overexpressed endothelial proteins. GFP‐tagged CD9, CD151, MDR1, EPORsh, TNFR1sh, TNFR2sh, HRH1, VAP‐1 (green) as well as untagged LOX1 expressed in CHO‐745 cells were labelled with respective antibodies (red). No antibodies were available which are directed against any of the nine extracellular domains of MRP2. Nuclei were stained with DAPI (blue). n.d., not determined.
