# Supplementary figures and images for: Evidence of promiscuous endothelial binding by Plasmodium falciparum‐infected erythrocytes
Source: Cell Microbiol. 2014 Feb 24;16(5):701–8. doi: 10.1111/cmi.12270 (PMC4114535; doi:10.1111/cmi.12270)

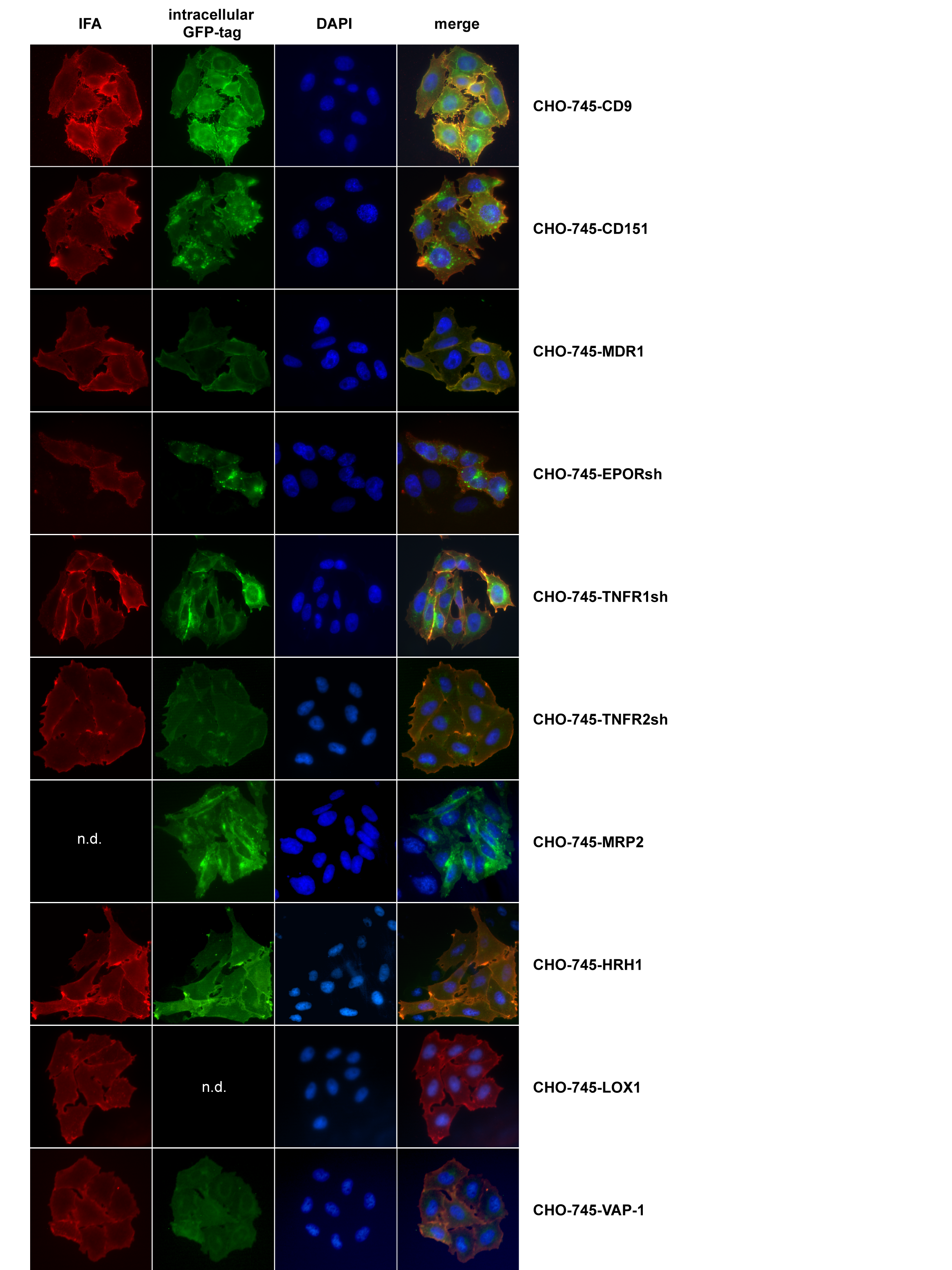

Supplement: Supplementary file 1 — Fig. S1. Antibody staining of recombinant proteins expressed on the surface of CHO‐745 cells. Immunofluorescence analyses (IFA) with non‐permeabilized CHO‐745 cells confirm surface localization of the overexpressed endothelial proteins. GFP‐tagged CD9, CD151, MDR1, EPORsh, TNFR1sh, TNFR2sh, HRH1, VAP‐1 (green) as well as untagged LOX1 expressed in CHO‐745 cells were labelled with respective antibodies (red). No antibodies were available which are directed against any of the nine extracellular domains of MRP2. Nuclei were stained with DAPI (blue). n.d., not determined. [file cmi-16-701-s3.tif]
